# Differences in Phosphorylated Histone H2AX Foci Formation and Removal of Cells Exposed to Low and High Linear Energy Transfer Radiation

**DOI:** 10.2174/138920212802510501

**Published:** 2012-09

**Authors:** Thomas Ernst Schmid, Olga Zlobinskaya, Gabriele Multhoff

**Affiliations:** 1 Klinikum rechts der Isar, Department of Radiation Oncology, Technische Universität München, D-81675 München, Germany; 2 CCG- Innate Immunity in Tumor Biology, Helmholtz Centre Munich (HMGU), Munich Germany

**Keywords:** DNA double strand breaks, Linear energy transfer, Radiation, γ-H2AX foci.

## Abstract

The use of particle ion beams in cancer radiotherapy has a long history. Today, beams of protons or heavy ions, predominantly carbon ions, can be accelerated to precisely calculated energies which can be accurately targeted to tumors. This particle therapy works by damaging the DNA of tissue cells, ultimately causing their death. Among the different types of DNA lesions, the formation of DNA double strand breaks is considered to be the most relevant of deleterious damages of ionizing radiation in cells. It is well-known that the extremely large localized energy deposition can lead to complex types of DNA double strand breaks. These effects can lead to cell death, mutations, genomic instability, or carcinogenesis. Complex double strand breaks can increase the probability of mis-rejoining by NHEJ. As a consequence differences in the repair kinetics following high and low LET irradiation qualities are attributed mainly to quantitative differences in their contributions of the fast and slow repair component. In general, there is a higher contribution of the slow component of DNA double strand repair after exposure to high LET radiation, which is thought to reflect the increased amount of complex DNA double strand breaks. These can be accurately measured by the γ-H2AX assay, because the number of phosphorylated H2AX foci correlates well with the number of double strand breaks induced by low or / and high LET radiation.

## INTRODUCTION

Particle therapy using carbon-ions or protons is becoming an important therapy concept worldwide [1]. Current and planned radiation therapy strategies use carbon ions to effectively treat malignant tumors [2, 3]. Compared with photons, the in-depth dose distribution of particles allows a more accurate dose administration, resulting in an increased therapeutic ratio. A major reason for the physical selectivity of the inverted dose profile is a sharp longitudinal dose drop at the end of the particle range. For heavy particles such as carbon ions lateral scattering can be neglected [4]. The increased therapeutic ratio permits dose escalation within the tumor which might result in a better tumor control. Another advantage is that high linear energy transfer radiotherapy comprises an increased radiobiological efficacy [5]. Carbon ions have been proven effective regardless of p53-status in gliomas, human tongue and lung cancer cell lines [6-8] and seem to be also beneficial in hypoxic tumors, as they induce an accelerated reoxygenation in comparison with X-rays [6, 7]. In fact, carbon ion radiotherapy has the potential of broaden the spectrum of primary radiotherapy, as first reports on favorable results for "radio-resistant tumors" like primary renal cell carcinoma have become available [6] and particle irradiation was shown to suppress metastatic potential of cancer cells [7].

An important prerequisite for a better understanding of such high-LET radiation effects on the DNA is the mechanistic description of the processing of DNA double strand breaks (DSBs). Ionizing radiation induces a variety of DNA lesions, including single and double strand breaks, DNA-protein cross-links and various base damages [7]. DSBs are induced linearly with radiation dose, with a yield of approximately 20-40 per cell nucleus and per Gy of X- or γ-rays [8]. DSBs cause most serious insults in cells because they can result in loss or rearrangement of genetic information, leading to cell death and carcinogenesis [9]. DSBs can be induced in the genome of eukaryotic cells by endogenous processes associated with oxidative metabolism, errors during DNA replication and various forms of site specific DNA recombination, as well as by exogenous agents such as ionizing radiation and chemicals [15].

It is well established that a localized deposition of high-LET particles result in complex DSBs [10] that cause cell death, mutations, genomic instability, and carcinogenesis. In contrast, the effects that are associated with repair of high-LET induced DSBs are not fully understood. Theoretical analysis and experimental evidence suggest an increased complexity and severity of complex DNA damage with increasing LET [11]. In general, DSBs in the DNA of higher eukaryotes induced by endogenous processes or exogenous agents are repaired either by non-homologous endjoining (NHEJ) or by homologous directed repair (HDR). An assignment of specific tasks for each of the two repair mechanisms is shown by the observation that defined DSB are repaired by either NHEJ or HDR [12]. 

Exposure to ionizing radiation cannot be avoided in the modern Western society. Ionizing irradiation can lead to a variety of deleterious effects including cancer and birth defects. Therefore, reliable, reproducible and sensitive biological and physical tests are required to assess the effects of radiation exposures on living organisms [13]. Herein, we discuss the usefulness of the DNA damage marker γ-H2AX for this purpose. Foci of γ-H2AX are formed in response to radiation-induced DNA double strand breaks and can be quantified by immunofluorescence microscopy or by flow- or laser-scanning cytometry [8]. The estimation of biological effects of low dose radiation effects requires the quantification of dicentrics and micronuclei (‘biological dosimetry’) because clinically relevant biological damage is predominantly reflected by chromosomal damage. Recently, new biological methods have been established based on molecular markers as indicators for irradiation [14]. These assays which can detect single- or double-strand DNA breaks, base damage, damage clustering or consequences derived thereof such as apoptosis, may have the potential to serve as biomarkers for detecting exposure of biological material to low dose irradiation [14]. In recent studies, Loebrich *et al. *(2005) as well as Rothkamm *et al. *(2007) have demonstrated that the assessment of *γ *-H2AX foci formation in human lymphocytes can also serve as a relevant biomarker for ionizing irradiation to a whole organism [8, 15]. (Table **1**) provides a concise comparison of previous and state-of-the art techniques to assess radiation damage tests and risk assessment.

### γ-H2AX Foci as a Marker of DNA Double Strand Breaks

A mechanistic description of the processing of DNA DSBs is important for the understanding of ionizing radiation effects leading to cell death, mutation, genomic instability, and carcinogenesis [16]. DNA damage sensing proteins have been shown to localize to the sites of DNA double strand breaks (DSB) within seconds to minutes following ionizing radiation (IR) exposure, resulting in the formation of microscopically visible nuclear domains referred to as radiation-induced foci [17]. Phosphorylation of H2AX provides an ideal functional marker to measure the induction of DSBs caused by radiation [18]. Using immunofluorescence staining and microscopy, γ-H2AX appears as distinct ionizing radiation-induced foci around the DSB [19] with an average early size of 0.2 μm2 indicating the rapid phosphorylation of thousands γ-H2AX molecules in domains of approximately 2 Mbp [31]. The number of γ-H2AX foci formed in this manner has been shown to be directly proportional to the number of DSBs formed, and their dephosphorylation has been found to correlate with repair of DSBs [20]. Given that on average 0.2-0.4 foci are induced per 10 mGy per cell, the γ-H2AX foci assay is capable of detecting radiation doses down to a few mGy under these conditions [8]. Interestingly, also a correlation between a loss of γ-H2AX foci and radiation sensitivity has been noted [20]. Additionally, it was shown that the rate of disappearance of radiation-induced γ-H2AX correlates directly with the rate of DNA repair at low levels of DNA damage, namely if fewer than 150 DSBs per genome (which approximately corresponds to 6 Gy of X-rays) are generated [18]. 

### DNA Double Strand Break Repair

DNA double strand breaks, if not repaired, may lead either to incorrect segregation during mitosis or to chromosomal loss [22]. DSB repair in mammalian cells seems to proceed through two genetically different pathways [21], homologous recombination [22] and non homologous end joining (NHEJ) [17, 22, 23]. These two pathways are biochemically distinct, have different substrate requirements, operate with different kinetics, and are used differently throughout the cell cycle [24]. NHEJ is much faster than HR [22] and is the primary pathway for DSB repair in eukaryotic cells [23]. Defects in NHEJ increase radiation sensitivity and the risk of carcinogenesis [24]. Rejoining of DNA ends by NHEJ requires little or no sequence homology and can occur in all stages of the cell cycle [19]. The repair by HR requires extensive sequence homology, and the repair process is important mainly during late S and G_2_ phases of the cell cycle [25]. The process of repair by HR or NHEJ may be controlled by the position of the cells in the cell cycle and in addition by the complexity of the damaged DNA ends. Less complex DSBs are repaired preferentially by NHEJ, which is the dominant pathway for repair of DSBs in mammalian cells during all stages of the cell cycle, while more complex DSBs containing multiple damaged sites that could not be repaired by NHEJ are repaired by HR when homologous DNA strands become available in late S and G_2_ phase [25]. 

### Scoring of the γ-H2AX Foci Number

Phosphorylated H2AX forms microscopically visible foci and the number of phosphorylated H2AX foci correlates well with the number of DSB induced by low-LET radiation [26]. The γ-H2AX focus labels the damage immediately after the induction of DNA DSBs and induces the cellular repair machinery. In Fig. (**1**), the appearance of HeLa cells with the immunofluorescence γ-H2AX foci pattern along the tracks of carbon ions with one carbon ion each in a 5x5 µm matrix and after 1,7Gy 200 kV x-ray is demonstrated. Although direct visualization of γ-H2AX is probably the most specific and efficient technique for counting DSBs in cells, it is a time consuming method [7]. The main disadvantages of foci scoring using fluorescence microscopy are the highly dynamic changes in foci numbers early after irradiation and difficulties associated with the actual scoring process which, if done by eye is somewhat subjective if slides are not coded [8]. 

Automated analysis can be used for the quantification of the γ-H2AX foci. The automated techniques can be performed in a consistent and reproducible manner and should not be compromised by investigator-introduced bias [27]. However, it should be taken into account that this method seems to be partly dependent on the respective thresholds or gating values used. Thus there is always the possibility that the present γ-H2AX data is affected by the automated quantification method [27]. Especially, at ≥ 2 Gy there is potential overlap of adjacent foci which may not be accurately separated resulting in an underestimation of foci counts. However, the influence on counted foci number should be the same when comparing different irradiation qualities. Therefore, the relative number should not be affected by this systematic error [33]. Similar to manual counting, overexposed cells with pan-nuclear γ-H2AX staining should be excluded from the analysis. According to MacPhail* et al*. [28] some of these cells were in S-Phase and G2 phase of the cell cycle.

In general, reliable foci scoring is limited to foci levels of less than about 20 by software counting to 50 (by visual counting) per lymphocyte, using conventional wide-field fluorescence microscopes [8]. Overlapping foci edges in all three dimensions at higher damage levels result in “underscoring”.

### Measuring γ-H2AX Intensity by Flow-Cytometry and Laser-Scanning-Cytometry

While fluorescence microscopy enables the detailed imaging of individual γ-H2AX foci, flow-cytometry and laser-scanning cytometry provide a rapid and large-scale method to quantify γ-H2AX by measuring the total fluorescence intensity in a high number of cells [8].

The group of Olive *et al.* [28, 29] pioneered the assessment of γ-H2AX phosphorylation by flow-cytometry to detect and measure DNA damage induced by X-rays. They could quantify the induction of γ-H2AX with a dose as low as 0.2 Gy of X-rays [28, 29]. The half-times of disappearance of the radiation-induced γ-H2AX ranging from 1.6 to 7.2 h were associated with a decrease in the number of foci, and were correlated with clonogenic survival for 10 cell lines. Several studies have reported linear relationships between γ-H2AX foci numbers and relative γ-H2AX fluorescence [30, 31]. Additionally, at doses from 2 to 16 Gy of X-rays a linear correlation was also seen between the γ-H2AX total intensity measured by flow-cytometry and the frequency of microscopic foci detected with image analysis [33]. It is known that the expression of γ-H2AX protein in response to the induction of DNA DSB is a kinetic event, which occurs within minutes and subsides due to its dephosphorylation [30]. Recently, it was also reported that cytometric assessment of γ-H2AX fluorescence in blood cells of X-irradiated patients offers a sensitive measure of DNA damage *in vivo* [26]. These authors stated that cytometric assessment of γ-H2AX expression is 100-fold more sensitive in detecting X-ray induced DNA damage [26] than the Comet assay [31], which can also be used to quantify DNA DSBs. The intensity of the γ-H2AX immunofluorescence of an individual cell corresponds very well to the extent of DNA damage in the cell nucleus. 

The laser scanning cytometer (LSC) combines a flow cytometer with a static image cytometer. Quantitative analysis by LSC is a method that provides equivalent data to that of a flow cytometer in a slide-based format. Laser scanning cytometry offers the possibility to rapidly quantify γ-H2AX immunofluorecence in large cell populations [32-34]. Moreover, it was shown that the LSC approach to measure γ-H2AX immunofluorescence is more sensitive compared with the alternative, commonly used foci scoring [35, 36]. The study of Whalen *et al.* showed a comparison of the number of γ-H2AX foci detected microscopically and by flow cytometry after iron ion exposure. Foci levels for γ-H2AX were significant over baseline levels for doses as low as 0.05Gy [36]. Laser-scanning cytometry and flow-cytometry both offer the advantage of speed, and the ability to resolve subpopulations based on expression of moieties that bind other fluorescence-tagged antibodies or molecules [28]. 

Although there are several advantages to use cytometry for quantifying γ-H2AX, there are some limitations that should be considered. The absolute intensity of γ-H2AX antibody binding per cell is dependent on the number of DSBs, the relative proportion of H2AX substrate and the H2AX kinase activity of the cell; all of which can vary [44]. The higher background in S/G_2_-phase cells is responsible for a two- to threefold reduction in the sensitivity for detecting DSBs in these cell populations [37]. Additionally, an interpretation of γ-H2AX intensity by flow cytometry as indicative of the presence of DSBs is complicated by the appearance of foci both in early apoptotic cells and in micronuclei [28]. However, most of these limitations are caused by biological variances which will also affect manual foci scoring. Higher numbers of analyzed cells and hence a higher confidence level of the obtained data as well as the higher throughput makes cytometry a valuable tool for analyzing the repair kinetics of radiation induced DSBs. Schmid and coworkers [34] have demonstrated that the slope of the dose–response curves was steeper for G_1_-phase cells relative to that for S+G2-phase cells. This difference is mainly based on the lower level of γ-H2AX expression in unirradiated G_1_-phase cells [28].

At low irradiation doses the scoring of foci by microscopical analysis is superior to that of flow- and laser-cytometric analysis of γ-H2AX with respect to sensitivity. This aspect limits the sensitivity of cytometric γ-H2AX analysis to a dose range which is greater than 100 mGy [8].

### DNA Double Strand Repair After Low LET Radiation Exposure

Most data on the loss of γ-H2AX foci with time using as an indicator of repair of DSBs in cells or cell lines have been obtained with low-LET radiation qualities. Several studies [21, 34, 35, 46] have reported linear relationships between foci numbers or relative γ-H2AX fluorescence and dose using X-rays but generally at high doses. Using a series of cancer cell lines, it was concluded that the presence of γ-H2AX foci at long times after γ-irradiation of exponentially growing monolayers may not always signify the presence of a DSB [38].

However, since the quantification of DSBs in human cells performed in these studies could be a basis for a sensitive biological dosimeter after a radiation accident, the question has been arisen, at which time point after radiation exposure the γ-H2AX foci should be counted [39]. For example, Rothkamm *et al*. [44] observed biphasic repair kinetics of DSBs with a fast earlier loss (50% during the first half hour) following by a slower loss (over several hours) of the remaining γ-H2AX foci signal in human lymphocytes after CT scans. Further qualitative analyses of γ-H2AX foci formation in normal human fibroblasts induced by silicon (54 keV/µm) or iron (176 keV/µm) have provided new insights into DNA damage processing kinetics, including evidence of increased clustering of DNA damage and slower processing with increasing LET [40].

### Differences in DNA Double Strand Repair After Low and High LET Radiation Exposure

Evidence has been accumulated that indicates that high-LET radiation induces complex DNA damage, a class of DNA lesions that includes two or more individual types of lesions within one or two helical turns of the DNA [22]. These lesions can be associated to the basic back bone, damaged bases, single strand breaks, or double strand breaks. There is convincing evidence that complex DNA lesions after high-LET radiation are more difficult to repair than isolated lesions and sometimes these lesions are irreparable [22]. Previous data indicate that DSBs which are repaired with a slow kinetics are localized predominantly in the periphery of the heterochromatin. Therefore, it was assumed that chromatin complexity may confer slow DSB repair kinetics [41]. In contrast, Jakob and coworkers have demonstrated that DSBs in heterochromatin in mammalian cells can induce phosphorylation of H2AX and a fast recruitment of repair proteins. [19]. The complexity of DNA lesions could determine the speed of the repair [17].

After high-LET radiation, a much lower number of DSB sites along an ion track is observed than expected on the basis of calculations due to fast clustering of DSBs, possibly to form repair factories. The organization of the chromatin and the track structure both affect the energy deposition of high-atomic-number and -energy (HZE) particles and lead to more clustered and non-randomly distributed DNA damage than was observed for low-LET radiation [42]. In the study of Karlsson and Stenerlow [43] the influence of LET on DNA DSBs in fibroblasts was determined after irradiation with photons or nitrogen-ions. With increasing LET, the number of induced DSB per track traversal and the complexity of the break were suggested to increase, leading to a decreased reparability of the damaged site. Closely spaced multiple DSBs could inhibit the attachment of repair proteins to other nearby DSBs, and this possibility increases with the ionization density or LET of the radiation quality [20]. Complex, non-randomly distributed DNA damage represents a considerable obstacle to efficient repair compared to free adducts and it has been shown that iron-ion-induced clustered lesions prevent efficient KU70/80 binding which is necessary for the NHEJ repair pathway [24].

An investigation of the induction and rejoining of DSBs in Chinese hamster cells and human fibroblasts using immunohistochemistry and quantification by image analysis has shown that at 6 h after exposure to α-particles (mean energy of 3.31 MeV) the induced γ-H2AX foci formation level remained significantly higher than that of γ-irradiated cells with isoeffective doses [44]. This study verified that quantification of the induction of γ-H2AX foci by different radiation qualities (γ-rays and α-particles) provides a method to undertake low dose studies. The study demonstrated that the induction of γ-H2AX foci increases with dose for both radiations and loss of γ-H2AX foci is a reasonable indicator of the timescale of rejoining of DSB induced by low LET radiation but less appropriate for those induced by high LET radiation.

Since the high complexity of such high-LET induced DSBs results in a retardation of DSB rejoining [45], the repair of DSB is much slower after high relative to low LET radiation [49, 50]. These results are well in line with findings of Hofman-Huther *et al*. (2004) who observed that the frequencies of cells containing complex chromosomal aberrations increased dramatically after irradiation of radio-resistant tumor cells with carbon ions versus X-rays [46]. One decade earlier, it was demonstrated that dense ionizing radiation, such as heavy ions, is much more efficient than sparsely ionizing radiation in the induction of complex-type exchanges [47]. These results have been recently confirmed by using the novel mFISH technique, where all 23 chromosome pairs can be painted in different colors. While X-rays induce very few complexes at doses below 2 Gy [48], most of the chromosome exchanges induced by alpha-particles [54], neutrons [49], or heavy ions [50] are indeed complex even at low doses. Examining X-ray and carbon ion induced effects on DNA in head carcinoma cells by using PCR of heterozygosity, Yamamoto *et al. *observed that most of the X-ray induced DNA damage occurred in the target region on one of the homologous chromosomes, whereas carbon ions caused homo-deletion, i.e. deletion of the counterparts in both homologous chromosomes [51].

The study of Schmid *et al. *[25] showed quantitatively the time course of the γ-H2AX-immunofluorescence intensity induced by high-LET (carbon ions) and low-LET radiation (X-rays). The time-effect relationship was best fitted by a bi-exponential function with corresponding half-life values of 24 ± 4 minutes and 13.9 ± 0.7 hours, respectively. These values were found to be independent of the LET of the analyzed radiation qualities. However, over the time course of 48 hours, the slow component was responsible for 80% of DNA DSB repair after cell exposure to carbon ions, but only for 47% of DNA DSB repair following X-ray irradiation. An explanation for this finding could be the high complexity of such carbon ion induced DSBs, which results in a retardation of DSB rejoining. Fig. (**2**) shows the integrated γ-H2AX fluorescence intensity over time for three different radiation qualities: alpha-irradiation, carbon ions and X-rays. Within the accuracy of measurement the fits lead to the same fast and slow reduction components of the γ-H2AX signal for all three radiation qualities and the half-life values for the slow and the fast component were determined to be 13.9 ± 0.7 hours and 24 ± 4 minutes, respectively.

It is known, that the high complexity of DSBs is a factor in the choice of repair pathway and homologous recombination is recruited in the repair of breaks with higher complexity during the late S and G_2_ phases of the cell cycle [25]. Heavy ion induced complex DSBs, which are in general more slowly repaired than X-ray induced breaks, are nearly always repaired by HR independent of chromatin localization. Thus it can be suggested that the speed of repair is an important factor determining the DSB repair pathway usage [12]. The spatially correlated DSBs after high-LET irradiation rejoin with slower kinetics and less completely than DSBs induced by low-LET radiation such as X-rays [42]. Less complex DSBs are repaired preferentially by NHEJ, which is the dominant pathway for repair of DSBs in mammalian cells during all stages of the cell cycle. 

One decade ago, Fowler [52] suggested the hypothesis to fit results on repair of DNA strand break damage, which may be relevant to recovery in mammalian tissues, by a binary, as well as or instead of monoexponential order. In fact, he demonstrated that such a second-order process with a single time parameter could explain the data indicating “apparently slowing down” repair previously fitted by multiexponential formulae requiring more time parameters. In accordance to recent findings that the DSB repair in mammalian cells seems to proceed through two genetically separate pathways the bi-exponential function may be still an incomplete description of the repair time course, but is probably more appropriate than other approximations like mono-exponential functions, which have been employed before [31].

## CONCLUDING REMARKS

In summary, radiation-induced γ-H2AX foci can be detected accurately after low and high LET irradiation. The process of repair by HR or NHEJ is controlled by the position of cells in the cell cycle and in addition by the complexity of the DNA damage. Complex DSBs after high-LET irradiation can increase the risk of mis-rejoining by NHEJ. Differences in the repair kinetics following high and low LET irradiation are mainly attributed to quantitative differences in their contributions of the fast and slow repair component. In general, there is a higher contribution of the slow component of DNA double strand repair after exposure to high LET irradiation, which is thought to reflect the increased amount of complex DNA double strand breaks.

## Figures and Tables

**Fig. (1):**
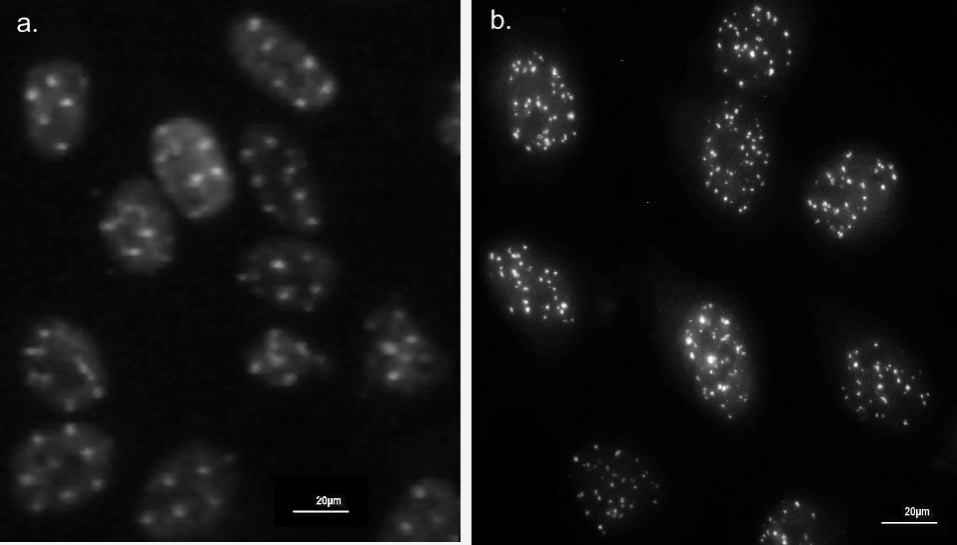
Representative view of HeLa cells stained with immunofluorescence labled antibody directed against γ-H2AX. Track of foci 15 min after irradiation with 55 MeV carbon ions (1.7 Gy) in a 5x5 µm^2^ matrix (a) and with 200 kV x-ray (1.7 Gy) (b). The nuclei of the cells were counterstained with propidiumiodide.

**Fig. (2):**
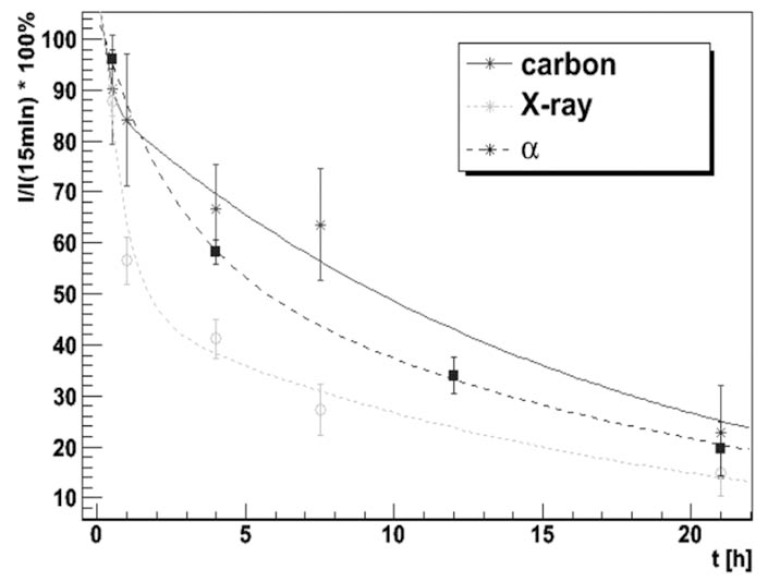
Integrated γ-H2AX fluorescence intensity over time, relative to values at 15 min after irradiation, fitted by a bi-exponential decrease function with fast and slow components for carbon ions (55 MeV), protons (20 MeV) and x-ray (200 kV). For this experiment HeLa cells were seeded in irradiation containers and allowed to adhere for 12 h. The culture technique and the irradiation conditions have been previously reported in detail [34].

**Table 1.** 

Technique	Damage Type	Minimal Dose	Requirements and Efforts	Advantages/Benefits	Drawbacks/Restrictions	References
Comet Assay	DNA DSB and SSB	200 mGy	Sophisticated Method Experience and Training is necessary	Detection of DNA strand damage in individual cells and in cell populations Widely accepted method	Cell Type Dependent (Not All Cell Types Work Good)	[53-54]
TUNEL Assay	Apoptosis	50 mGy	Especially useful for tissue sections Experience is obligate Cost effective	Works in frozen and in formalin-fixed, paraffin-embedded tissue sections. Samples can be stored for months before analyzing. Using DNA counter stain the phase of the cell cycle where apoptosis is occurring can be measured.	Detects DNA fragmentation (SSB and DSB) but it can not differentiate apoptosis from necrosis Fixation and handling of tissue can significantly alter the results of the TUNEL assay	[55-57]
Colony Survival Assay	Cell Survival	1 Gy	Easy method when all parameters, are known Takes at least two weeks and is time consuming	Gold standard Cell reproductive death after treatment with ionizing radiation	Only cell population can be studied Adherent growing cells necessary Large cell number needed Sterility issue	[58-62]
Annexin V	Apoptosis	50 mGy	Flow cytometer necessary Easy and very fast method for quantification	Can detect differences in cell death by necrosis or apoptosis Capable for live-cell imaging	Annexin V staining has to be performed on live cells Cell number is restricted	[63, 64]
Micronucleus Assay	Micronuclei	200 mGy	Widely accepted method Experience is essential Robust and reproducible Manual and automated scoring	Adherent and circulating cells, also isolates from tissue Biodosimetry of genotoxic exposures Fast assay permits screening of large numbers of cells.	Variable micronucleus background frequency Indirect measure of DNA damage	[65, 66]
Chromosome aberrations	Dicentric Chromosomes	20 mGy	Gold standard Experience is essential Robust and reproducible	Quantification of dicentrics remains the method of choice for estimating the effect of exposures to low dose levels of radiation e.g. biological dosimetry Because of the long life of some lymphocytes, chromosomal aberrations can be detected even years after exposure	Only late effects Need of a reliable reference curve Scoring needs a high level of experience	[14, 67]
γ-H2AX	DNA DSB	100 mGy	Robust and reproducible Very fast method Manual and automated scoring of foci Flow- and Laser-Scanning-Cytometry possible	New method for biodosimetry and predictive studies Adherent and circulating cells, tissues samples Different detection system are available	Limited to 3-4 Gy for foci scoring Background fluorescence can influence evaluation Evaluation needs experience Staining depends on cell cycle	[13, 15], [34, 68]

## ABBREVIATIONS

DSB: = DNA Double Strand Break
H2AX: = H2A Histone Family Member X
HR: = Homologous Recombination
LET: = Linear Energy Transfer
LSC: = Laser Scanning Cytometery
mFISH: = Multicolor Fluorescence in Situ Hybridization
NHEJ: = Non-Homologous End Joining
